# BioNanoAnalyst: a visualisation tool to assess genome assembly quality using BioNano data

**DOI:** 10.1186/s12859-017-1735-4

**Published:** 2017-06-30

**Authors:** Yuxuan Yuan, Philipp E. Bayer, Armin Scheben, Chon-Kit Kenneth Chan, David Edwards

**Affiliations:** School of Biological Sciences, the University of Western Australia, Perth, WA Australia

**Keywords:** BioNano, Misassembly, Restriction enzyme cut site, Optical map

## Abstract

**Background:**

Reference genome assemblies are valuable, as they provide insights into gene content, genetic evolution and domestication. The higher the quality of a reference genome assembly the more accurate the downstream analysis will be. During the last few years, major efforts have been made towards improving the quality of genome assemblies. However, erroneous and incomplete assemblies are still common. Complementary to DNA sequencing technologies, optical mapping has advanced genomic studies by facilitating the production of genome scaffolds and assessing structural variation. However, there are few tools available to comprehensively examine misassemblies in reference genome sequences using optical map data.

**Results:**

We present BioNanoAnalyst, a software package to examine genome assemblies based on restriction endonuclease cut sites and optical map data. A graphical user interface (GUI) allows users to assess reference genome sequences on different computer platforms without the requirement of programming knowledge. The zoom function makes visualisation convenient, while a GFF3 format output file gives an option to directly visualise questionable assembly regions by location and nucleotides following import into a local genome browser.

**Conclusions:**

BioNanoAnalyst is a tool to identify misassemblies in a reference genome sequence using optical map data. With the reported information, users can rapidly identify assembly errors and correct them using other software tools, which could facilitate an accurate downstream analysis.

**Electronic supplementary material:**

The online version of this article (doi:10.1186/s12859-017-1735-4) contains supplementary material, which is available to authorized users.

## Background

Reference genome assembly plays an important role in genomic studies, as it supports the analysis of genetic diversity, genome evolution and the genetic basis of heritable phenotypes. Since the advent of second generation sequencing (SGS), the number of available genome assemblies has constantly grown. Compared to Sanger sequencing, SGS technologies are faster, with higher throughput and lower costs [1]. However, due to the large number of repetitive regions in some genomes and the short length of sequencing reads, assemblies generated using SGS are often collapsed and fragmented [2]. To overcome these problems, long read sequencing such as produced by Pacific Biosciences and Oxford Nanopore have been applied. However, relatively high costs and error rates associated with these technologies have hampered their broad adoption [1, 3, 4]. In contrast to DNA sequencing, optical mapping uses the physical location of restriction endonuclease cut sites to assist genome scaffolding and structural variation detection. The average length of single physical maps is more than 200 Kb [5], substantially longer than any single molecule sequencing reads produced by commonly used sequencing platforms.

The most common optical mapping approach uses the BioNano Irys and has been applied to a wide range of organisms [6–11]. Among these studies, most used BioNano optical mapping to help genome scaffolding and for structural variation detection, and there are still no studies reported of genome misassembly identification and correction using BioNano data or use of this data to examine the reference genome assembly quality. Here we describe BioNanoAnalyst, an open-source software package to facilitate the quality assessment of genome assemblies using BioNano data. Written in Python and converted to system specific applications, BioNanoAnalyst can run on all common computing platforms with minimal dependencies. It offers a Graphical User Interface (GUI) to visualise the results in forms of tables showing the enzyme restriction site information, and graphs displaying the assembly qualities. BioNanoAnalyst can export the results in GFF3 format for incorporation in a genome browser to assess misassemblies at the nucleotide level. Based on this information, misassembly correction can be undertaken using other tools to improve the quality of genome assemblies.

## Implementation

**Fig. 1:**
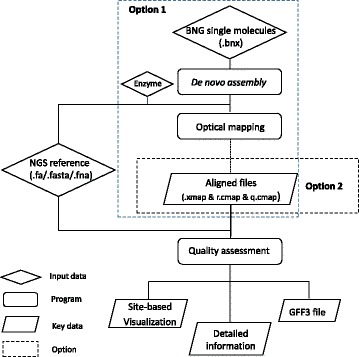
Pipeline used in BioNanoAnalyst. Two starting point options are available. Both options require the input of an NGS genome assembly

BioNanoAnalyst uses an assessment procedure based on a reference cmap file, query cmap file and the combined xmap file. The combined xmap file is obtained by a comparison between the reference cmap file and query cmap file using RefAligner. Two starting options are available in BioNanoAnalyst, using either raw data or previously aligned data (Fig. 1). The first option uses the raw data from BioNano platforms such as Irys to produce the assessment. In this case, BioNanoAnalyst follows the same steps as BioNano IrysView by using the executables RefAligner and Assembler to perform single molecule map *de novo* assembly and optical mapping. Parameter settings allow users to customise the software based on their available computing resource, such as the number of CPUs or size of requested memory. The second option uses previously aligned results in cmap and xmap format. Both options require the input of a reference genome sequence.

The confidence score is one of the assessment criteria introduced by BioNano Genomics to evaluate the alignment quality between a reference and query maps. A higher confidence score means a higher possibility to get a better alignment between matched adjacent enzyme restriction sites. In IrysView, the range of confidence score is 0–60 with a step size of 5, while in BioNanoAnalyst we allow any number > =0. Users can select their optimal confidence score depending on their mappings, and usually we recommend 10–20. If users select a large confidence score, the information with a confidence score below will be hidden in the xmap file. After specifying a confidence score and processing, reports are generated detailing the quality assessment.

### Tukey’s method to detect misassemblies

**Fig. 2:**
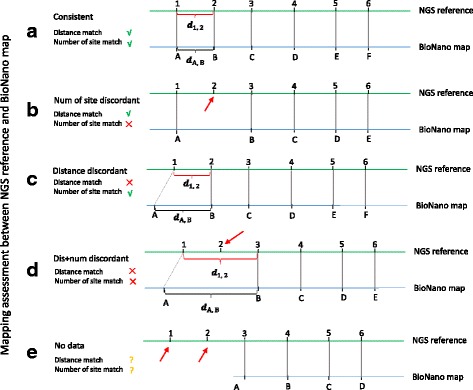
Examples to show how BioNanoAnalyst assesses the quality of NGS genome assembly. In the examples we assume that there are 6 selected enzyme cut sites (e.g. Nt.BspQI) on the NGS reference. Five different mapping cases are given to assist the understanding of the scoring in BioNanoAnalyst. **a** shows a good match between NGS reference and BioNano map, and BioNanoAnalyst gives score 4 to each restriction site on the NGS reference. **b** shows a conflict in the number of mapped restriction site between matched adjacent restriction sites. In this case, BioNanoAnanlyst assigns 3 to site 1–3. **c** shows a physical distance matching conflict (*diff* falls outside one of the boundaries) between mapped NGS reference and BioNano map. In this case, BioNano scores site 1 and 2 with score 2. **d** shows a physical distance matching conflicts and number of restriction site matching conflict between NGS reference and BioNano map. In this case, score 1 is given to site 1–3. **e** shows a case that there is no BioNano map mapping to some sites on the NGS reference, such as site 1 and 2. In this case BioNanoAnalyst gives score 0 to site 1 and 2

BioNanoAnalyst reports the assembly quality of the input reference sequences based on restriction site ID and distance differences between BioNano consensus maps and reference genome sequences. It does this by comparing pairs of restriction sites aligning between the assembly and BioNano maps. As an example (Fig. 2a), assume that the assembly sequence has 6 restriction sites from 1 to 6, and the BioNano map has 6 restriction sites from A to F, each restriction cut site has its own position (denoted as *P)*. In the alignment, (1, A), (2, B), (3, C), (4, D), (5, E) and (6, F) are matched. However, some cases such as restriction site 2 in the assembly in Fig. 2b have no BioNano data match, so BioNanoAnalyst reports them as a questionable restriction site, which may not belong to that position and may be misplaced or cannot be captured owing to a low coverage of the BioNano map or DNA double-strand breaks in that position leading to an enzyme cut site missing in the BioNano map [12].

In a perfect assembly, the absolute distance between restriction site 1 and 2 (*d*_*1, 2*_ = |*P*_*2*_ *–P*_*1*_|, *d* means distance, *d*_*1, 2*_ means distance between restriction 1 and 2), 2 and 3, 3 and 4, 4 and 5, and 5 and 6 should be the same as the distance between restriction site A and B, B and C, C and D, D and E, and E and F respectively (normalized) (Fig. 2a). However, noise from the BioNano consensus maps and the genome assembly influences the relative difference between restriction sites. Misassemblies can increase the differences between calculated distances and affect their statistical distribution, preventing them from following a normal distribution or skewing the normal distribution. To find significant differences between distances, we use Tukey’s method [13] to report questionable assembly regions in the reference genome sequence by identifying distance-difference outliers.

For each pair of restriction site pairs, the difference in distance is recorded as *diff* (e.g. *diff*_*1*_ = *d*_*1, 2*_ - *d*_*A, B*_ in Fig. 2a), and all *diffs* of all pairs are sorted to calculate the first and third quartile. Based on the first quartile (Q1) and third quartile (Q3), the “lower boundary” (2.5Q1–1.5Q3) and “upper boundary” (2.5Q3–1.5Q1) are calculated. If *diff* falls within the lower and upper boundary, the alignment is counted as valid and the assembly agrees with the BioNano map. If *diff* is outside the upper or lower boundary, the region is classed as a candidate misassembly. When *diff* < lower boundary this means there is sequence information missing in the reference genome, and when *diff* > upper boundary this indicates that there is additional sequence information in the reference genome. A complex case occurs when *diff* is outside the upper or lower boundary and the region contains one or more restriction sites without a match in the BioNano map. For example, assuming there is a significant difference between *d*_1, 3_ and *d*_A, B_ in Fig. 2d, we mark this case as a restriction site id- and position- matching problem between restriction site 1 and 3 on the NGS reference, and it has a high potential of contig misplacement between restriction site 1 and 3.

### Scoring each restriction endonuclease cut site

BioNanoAnalyst divides the restriction endonuclease cut sites on the reference into five quality groups with a numerical score assigned to each. Quality scoring is based on the consistency between the BioNano map and the reference, which is evaluated using the *diff* and matched number of restriction sites in the two assemblies (Fig. 2). By comparing these, restriction sites are assigned a quality score from 4 to 0. Score 4 is given when there are no *diff* and number of restriction site conflicts between matched BioNano map and reference, such as all restriction sites in Fig. 2a. Score 3 is assigned when *diff* is consistent between assemblies but the number of restriction sites in the mapped regions is in conflict, for instance restriction site 1–3 in Fig. 2b. Score 2 indicates that there is only distance conflict between restriction sites and *diff* is outside the boundaries, such as restriction sites 1 and 2 in Fig. 2c. Restriction sites are assigned a score of 1 when they have both distance conflict (*diff* falls outside the boundaries) and number of restriction site conflict between matched regions, for example restriction site 1–3 in Fig. 2d. A score of 0 means that there is no BioNano data mapping to those restriction site regions in the reference and the restriction site is not involved in any condition which has already been described, such as restriction 1 and 2 in Fig. 2e. This can be caused by low coverage of BioNano maps or misassembly in the reference. Scores are displayed in a double y-axis plot with the coverage of corresponding BioNano data on the left y-axis and score on the right y-axis. Users can verify the accuracy of the assessment from BioNanoAnalyst by locating the position and coverage of the restriction cut site. The coverage comes from the restriction site on the query maps. If the query maps have no restriction site mapping to the corresponding site on the reference, there will no coverage information showing on the mapping canvas. If the coverage of a restriction site is high (> the average) and BioNanoAnalyst reports the restriction site is a questionable restriction site, it highly suggests that the assessment by BioNanoAnalyst is correct. If the coverage of the restriction site is lower than the average, we suggest checking the quality score first. If the quality score is less than 10, other method may be needed to check the report from BioNanoAnalyst. If the quality score is larger than 10, it highly suggests that the assessment from BioNanoAnalyst is correct.

## Results

### Visualization

**Fig. 3:**
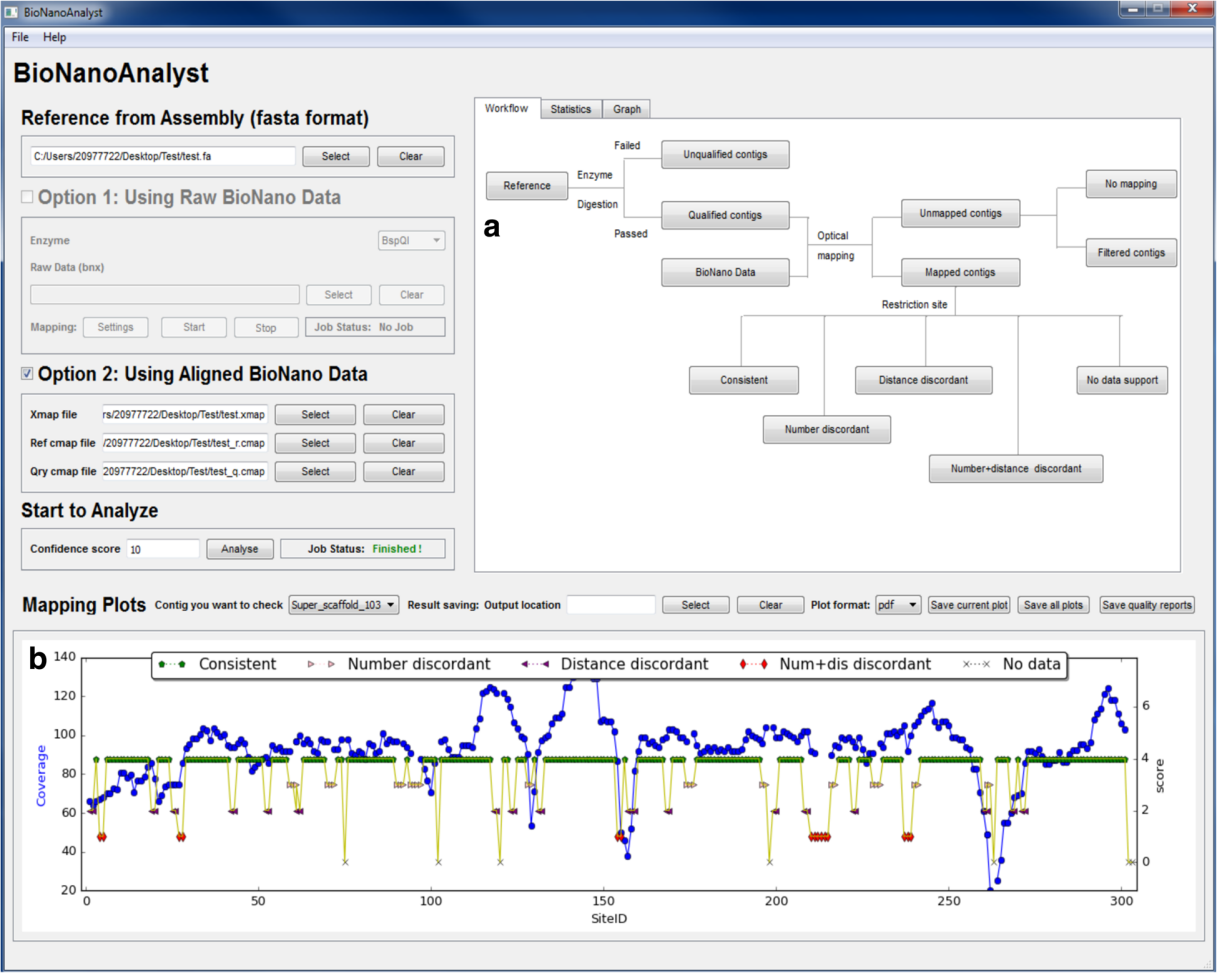
A screenshot of the graphical user interface of BioNanoAnalyst. Once the analysis if finish, users can click the buttons on the ‘Workflow’ canvas to check the corresponding restriction site ID and location (**a**). Users can also select a contig from the drop-down box to visualise the results (**b**). In the double y plot, users can click and zoom on each restriction site to view its id and location on the reference genome. The figure was generated using the first public subterranean clover genome assembly

**Fig. 4:**
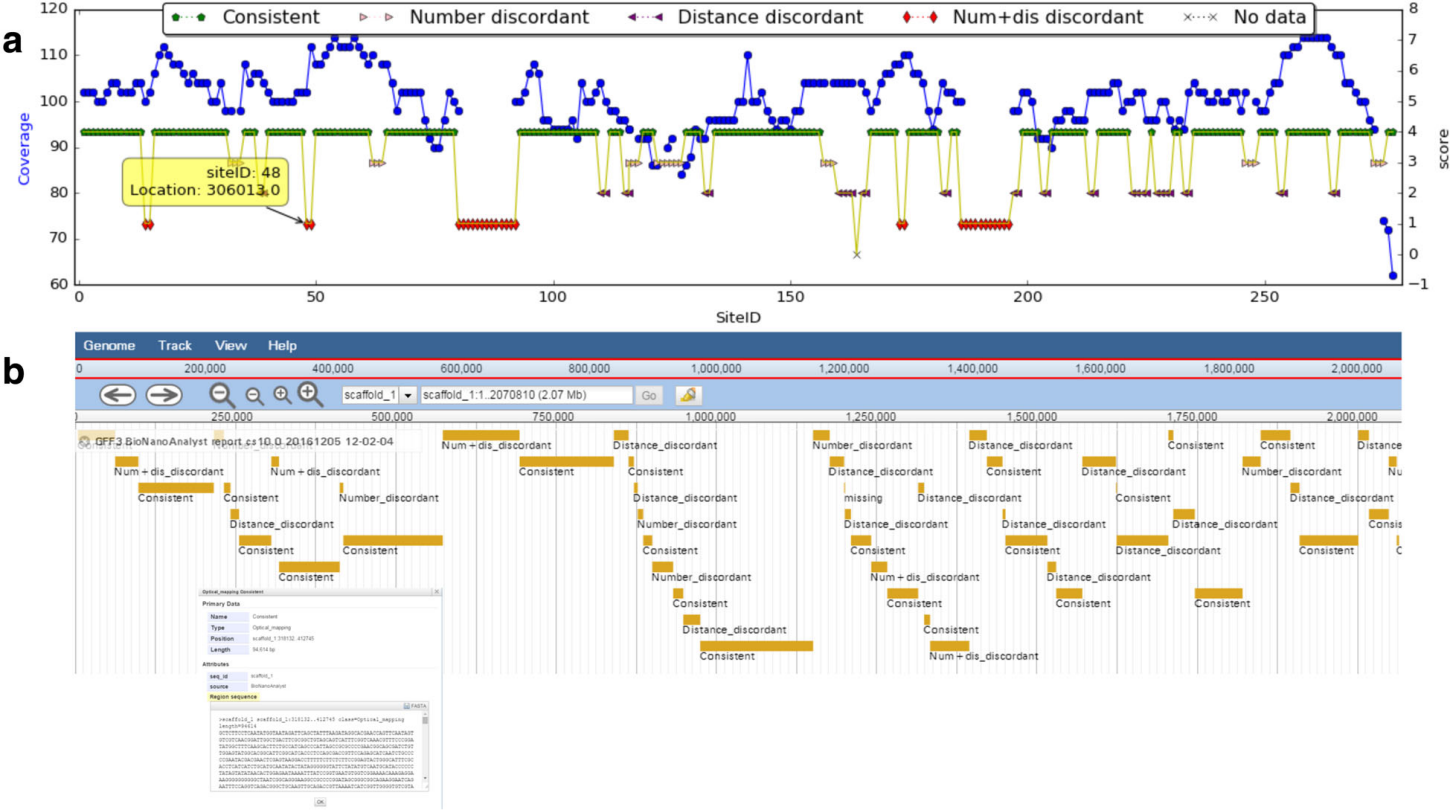
Misassembly visualisation in BioNanoAnalyst (**a**) and a JBrowse genome browser (**b**). The upper figure (**a**) is generated by BioNanoAnalyst, which can be shown in the ‘Mapping plots’ canvas. The lower figure (**b**) is a screenshot from JBrowse presenting the GFF3 file generated by BioNanoAnalyst. In this browser, users can right click the mouse to check the sequence of misassemblies. The data used to generate this figure was from the test data which has been provided in the Github repository

BioNanoAnalyst provides a GUI, with tables and graphs which the information from the initial enzyme digestion to the final mapping. All information is displayed by clicking the respective button on the “Workflow” canvas (Fig. 3a). The assembly quality of each reference sequence is displayed on the “Mapping plots” canvas (Fig. 3b). On this canvas, a double y plot assists misassembly assessment in terms of restriction site ID and location (Fig. 4a). In this plot, users can scroll to zoom and visualise specific restriction sites. Users can also click on a restriction site to view its ID and location on the reference genome. The GFF3 file generated by BioNanoAnalyst can be imported directly to a genome browser such as JBrowse [14], to visualise the location and nucleotide sequences of the predicted misassemblies (Fig. 4b).

### Computational requirement test

**Fig. 5:**
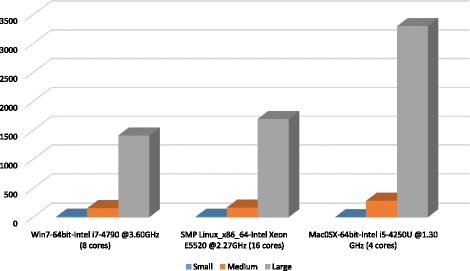
Performance of BioNanoAnalyst on different operating systems and computing configurations. The tested genome sizes are 5 Mb, 500 Mb and 1000 Mb. The Y axis indicates the running time (seconds) for different genome sizes. The X axis shows the computing configuration of different platforms tested

To test the running efficiency and platform requirements of BioNanoAnalyst, we used small (5 Mb), medium (500 Mb) and large genome sizes (1000 Mb) for three different species (*E. coli*, clover and *Brassica napus* canola) analysed on local Windows, Linux and MacOS platforms. The coverage of BioNano optical mapping data is over 70X. The selected enzyme density in the three genomes is around 11/100Kb. The analysis files used were xmap, r.cmap and q.cmap. The confidence score (10) was selected using the average confidence score of these three datasets. The computing configuration and test running time are shown in Fig. 5. During task processing, BioNanoAnalyst mostly used one CPU, and during specific stages, such as assigning a score to each restriction site, it used all available CPUs −1. The peak memory consumption was ~100 Mb, ~1 Gb and ~1.5 Gb for the three datasets respectively.

### Performance of the Tukey’s method

**Fig. 6:**
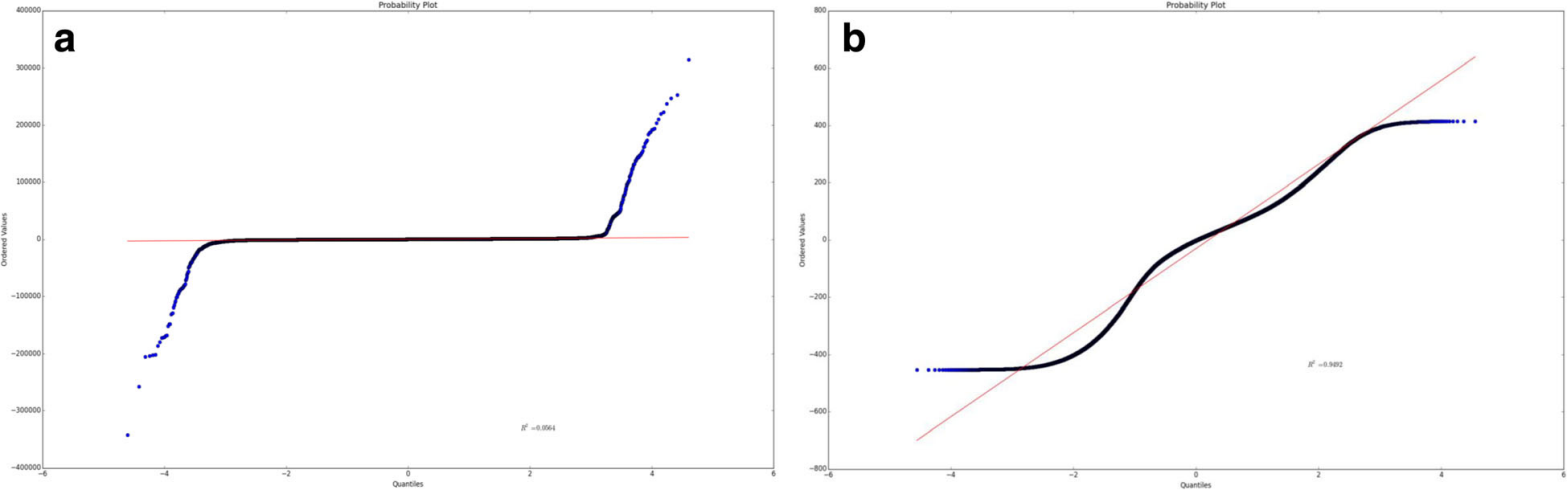
The distribution of *diffs* in NA12878 to hg19 with and without outlier trimming. Before outlier trimming (**a**), the R^2^=0.0564 which means that *diffs* don’t follow a normal distribution. After outlier trimming (**b**), R^2^=0.9492 which means that the remained *diffs* follow a normal distribution

To test the performance of method used in BioNanoAnalyst, we used the public NA12878 datasets and the hg19 human reference. After mapping using RefAligner, ~90% of the h19 reference was covered by NA12878 BioNano data. A distribution of all *diffs* calculated from the mappings between hg19 and NA12878 BioNano maps without outlier trimming is shown in Fig. 6a, with an R^2^=0.0564. After outlier trimming, the distribution shown in Fig. 6b had an R^2^=0.9492 (Additional file 1: Table S1).

The number of questionable restriction sites between NA12878 and hg19 has been shown in Table 1. As most human BioNano data is used to detect structure variations, we also used the table provided by BioNanoAnalyst to check indels in NA12878. When *diff* < 0, there is an insertion in NA12878, and when *diff* > 0, there is a deletion. In total, we find 165,180 Indels, which account for 94.5% of those benchmarked by Zook et al. [15]. Missing indels may exist in the BioNano data uncovered regions.

**Table 1:** Number of restriction sites for cases as shown in Fig. 2a-e between NA12878 and hg19, and between hg18 and hg19

Case	hg18_to_hg19	NA12878_to_hg19
Figure 2a	229,989	213,146
Figure 2b	83	18,544
Figure 2c	33,573	105,273
Figure 2d	29,437	5308
Figure 2e	61,144	13,665

### Accuracy test

**Fig. 7:**
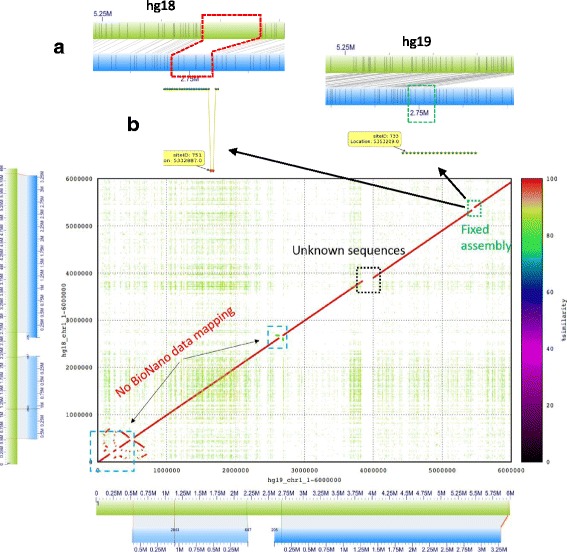
Validation of the findings from BioNanoAnalyst. The mummerplot shows the consistency of assembly from a nucleotide level. The BioNano optical mapping indicates the consistency of assembly in a motif level. From both comparisons, it can be seen BioNano optical mapping and mummerplot results can be consistent with each other. When validating the accuracy of the finding from BioNanoAnalyst (**b**), the BioNano optical mapping gives a support (**a**)

The human reference genome assembly is the most well assembled of the available large genomes, but continuous upgrades of this reference are ongoing. To validate the accuracy of the misassemblies detected by BioNanoAnalyst, we selected the public human BioNano genomic data Sample NA12891 and different versions of public human genome references (hg19 and hg18). We firstly used mummerplot [16] to show the differences between the assemblies. The enzyme used to generate the BioNano data was *Nt.BspQI* (GCTCTTCN). The confidence score (60) selected in this test was the maximum used in IrysView. An example output from BioNanoAnalyst is presented in Fig. 7, showing the consistency between the findings from BioNanAnalyst and mummerplot in the first 6 Mb of chromosome 1 on hg18 and hg19. In the mummerplot, apart from the unknown sequences (Ns) and the non-BioNano mapped regions, the biggest difference found is shown at position 5.35 Mb (Fixed assembly). When tracking back to the BioNanoAnalyst result on hg18 (Fig. 7a), we found that BioNanoAnalyst gave a ‘distance mapping problem’ report (Fig. 7b). When checking the same region in hg19, it shows that the error has been fixed and there is no misassembly report from BioNanoAnalyst.

To directly compare the differences between the human references hg18 and hg19, we digested both assemblies in silico with the enzyme *Nt.BspQI*. We used the generated hg19.cmap as the reference map and hg18.cmap as the query map and compared them using the RefAligner. After obtaining the xmap file, we analysed the data with BioNanoAnalyst and found that in hg19 there was 19.74 Mb nucleotide information missing from the hg18 and an additional 22.68 Mb of nucleotide information. The remaining matched sequences are the same with *diff* equal to 0 (Additional file 1: Table S2). Number of restriction sites reported by BioNanoAnalyst has been given in Table 1.

To test the false positive and false negative identification rates in the comparison between hg18 and hg19, we randomly selected 100 regions from the 247,580 BioNanoAnalyst reported consistent regions (*diff* == 0) in hg18 and hg19, and 100 regions from the 39,822 potentially modified regions (*diff*! = 0) in hg19 and hg18 and pairwise aligned them using BLASTN (v2.2.29+) [17]. The assessment criteria were from the default BLASTN results, which were percentage of identical matches, alignment length, number of mismatches and number of gap openings. We found that both the false positive rate and the false negative rate were 0. However, among the *diff*! = 0 regions, we found that 97% of those extracted hg19 sequences only added or deleted some nucleotides in either 5′ end or 3′ end compared to those sequences extracted from hg18. For the remaining 3% of sequences, they changed some information inside the 5′ and 3′ ends compared to those sequences in hg18.

## Discussion

BioNanoAnalyst uses hashtables to store information. The computational requirement test showed that BioNanoAnalyst is efficient in memory use with an acceptable running speed on a local computer. The performance of Tukey’s method used in BioNanoAnalyst was tested using public NA12878 and NA12891 BioNano datasets, and comparison between the human reference genome hg18 and hg19. Although a percentage of false positives and false negatives have been given based on analysis of human genome references, these numbers may vary depending on data used. Because BioNanoAnalyst uses the aligned result from RefAligner, the accuracy of BioNanoAnalyst can be affected by the performance of RefAligner. The quality of reference and query maps are also important for the analysis carried out in BioNanoAnalyst. During testing, the majority reported misassemblies have a distance-difference with BioNano consensus maps. The reason might be a poor resolution in the reference in repeat reconstruction.

BioNanoAnalyst is designed to detect misassembed regions in a reference genome. When assembly quality is high, such as in the human genome reference, BioNanoAnalyst can compare individuals to identify deletions or insertions in particular regions. However, as many non-human assemblies are not high quality, additional information is required to identify whether inconsistencies are caused by a deletion or insertion. In many non-human genomes, there is a high potential of misassembly in the reference through, for instance, collapsed repeats. As we use distance differences to detect misassembly, BioNanoAnalyst is not efficient in finding complex misassemblies such as false translocations and inversions, however the BioNanoAnalyst results table can be used to help assess false inversions.

## Conclusions

The BioNanoAnalyst package offers a simple way to assess genome assemblies using BioNano data, with fast run times for different genome sizes and on different platforms, detailed misassembly reports and standard GFF3 based visualisation. BioNanoAnalyst is a useful and unique tool to evaluate the quality of reference genome assemblies. The GUI provides a visual representation of the assembly using restriction site IDs and physical locations, enabling users to easily find misassembled regions. It also provides options for users to visualise and present misassemblies in GFF3 format using standard genome browsers such as JBrowse. The graphs and tables generated by the tool comprehensively show the locations and status of assemblies as classified. We believe that BioNanoAnalyst is a valuable tool for assessment of the quality of reference assemblies using BioNano data.

## Additional file

Additional file 1: Table S1. Mappings between NA12878 BioNano data and hg19. **Table S2.** Mappings between hg19 and hg18 using RefAligner. (XLSX 59183 kb)

## Abbreviations

CPU: Central processing unit
Diff: Physical distance difference between mapped adjacent enzyme restriction sites
GFF: Generic feature format
GUI: Graphical user interface
NGS: Next generation sequencing
Q1: First quartile
Q3: Third quartile
SGS: Second generation sequencing
SV: Structural variation

## References

[1] Goodwin S, McPherson JD, McCombie WR (2016). Coming of age: ten years of next-generation sequencing technologies. Nat Rev Genet.

[2] Alkan C, Sajjadian S, Eichler EE (2011). Limitations of next-generation genome sequence assembly. Nat Methods.

[3] Berlin K, Koren S, Chin CS, Drake JP, Landolin JM, Phillippy AM (2015). Assembling large genomes with single-molecule sequencing and locality-sensitive hashing. Nat Biotechnol.

[4] Koren S, Phillippy AM (2015). One chromosome, one contig: complete microbial genomes from long-read sequencing and assembly. Curr Opin Microbiol.

[5] Shelton JM, Coleman MC, Herndon N, Lu N, Lam ET, Anantharaman T, et al. Tools and pipelines for BioNano data: molecule assembly pipeline and FASTA super scaffolding tool. BMC Genomics. 2015;16:734. 10.1186/s12864-015-1911-8 PMC4587741 26416786

[6] Mostovoy Y, Levy-Sakin M, Lam J, Lam ET, Hastie AR, Marks P, et al. A hybrid approach for de novo human genome sequence assembly and phasing. Nat Methods. 2016;13(7):587–90. 10.1038/nmeth.3865 PMC4927370 27159086

[7] Pendleton M, Sebra R, Pang AW, Ummat A, Franzen O, Rausch T, et al. Assembly and diploid architecture of an individual human genome via single-molecule technologies. Nat Methods. 2015;12(8):780–6. 10.1038/nmeth.3454 PMC4646949 26121404

[8] Rosenfeld JA, Reeves D, Brugler MR, Narechania A, Simon S, Durrett R, et al. Genome assembly and geospatial phylogenomics of the bed bug *Cimex lectularius*. Nat Commun. 2016;7:10164. 10.1038/ncomms10164 PMC4740774 26836631

[9] Stankova H, Hastie AR, Chan S, Vrana J, Tulpova Z, Kubalakova M, et al. BioNano genome mapping of individual chromosomes supports physical mapping and sequence assembly in complex plant genomes. Plant Biotechnol J. 2016;14(7):1523–31. 10.1111/pbi.12513 PMC5066648 26801360

[10] VanBuren R, Bryant D, Edger PP, Tang H, Burgess D, Challabathula D, et al. Single-molecule sequencing of the desiccation-tolerant grass Oropetium thomaeum. Nature. 2015;527(7579):508–11. 10.1038/nature15714 26560029

[11] Xiao S, Li J, Ma F, Fang L, Xu S, Chen W, et al. Rapid construction of genome map for large yellow croaker (*Larimichthys crocea*) by the whole-genome mapping in BioNano Genomics Irys system. BMC Genomics. 2015;16:670. 10.1186/s12864-015-1871-z PMC4559010 26336087

[12] Hastie AR, Dong L, Smith A, Finklestein J, Lam ET, Huo N, et al. Rapid genome mapping in nanochannel arrays for highly complete and accurate de novo sequence assembly of the complex *Aegilops tauschii* genome. PLoS One. 2013;8(2):e55864. 10.1371/journal.pone.0055864 PMC3566107 23405223

[13] Tukey JW. Exploratory data analysis. Reading, MA: Addison-Wesley; 1977.

[14] Skinner ME, Uzilov AV, Stein LD, Mungall CJ, Holmes IH (2009). JBrowse: a next-generation genome browser. Genome Res.

[15] Zook JM, Chapman B, Wang J, Mittelman D, Hofmann O, Hide W, et al. Integrating human sequence data sets provides a resource of benchmark SNP and indel genotype calls. Nat Biotechnol. 2014;32(3):246–51. 10.1038/nbt.2835 24531798

[16] Kurtz S, Phillippy A, Delcher AL, Smoot M, Shumway M, Antonescu C, et al. Versatile and open software for comparing large genomes. Genome Biol. 2004;5(2):R12. 10.1186/gb-2004-5-2-r12 PMC395750 14759262

[17] Altschul SF, Gish W, Miller W, Myers EW, Lipman DJ (1990). Basic local alignment search tool. J Mol Biol.

